# RcsAB is a major repressor of *Yersinia* biofilm development through directly acting on *hmsCDE*, *hmsT*, and *hmsHFRS*

**DOI:** 10.1038/srep09566

**Published:** 2015-04-01

**Authors:** Nan Fang, Huiying Yang, Haihong Fang, Lei Liu, Yiquan Zhang, Li Wang, Yanping Han, Dongsheng Zhou, Ruifu Yang

**Affiliations:** 1 State Key Laboratory of Pathogen and Biosecurity, Beijing Institute of Microbiology and Epidemiology, Beijing 100071, China

## Abstract

Biofilm formation in flea gut is important for flea-borne transmission of *Yersinia pestis*. There are enhancing factors (HmsHFRS, HmsCDE, and HmsT) and inhibiting one (HmsP) for *Yersinia pestis* biofilm formation. The RcsAB regulatory complex acts as a repressor of *Yesinia* biofilm formation, and adaptive pseudogenization of *rcsA* promotes *Y. pestis* to evolve the ability of biofilm formation in fleas. In this study, we constructed a set of isogenic strains of *Y. pestis* biovar *Microtus*, namely WT (RscB+ and RcsA-), *c-rcsA* (RscB+ and RcsA+), *ΔrcsB* (RscB- and RcsA-), and *ΔrcsB/c-rcsA* (RscB- and RcsA+). The phenotypic assays confirmed that RcsB alone (but not RcsA alone) had an inhibiting effect on biofilm/c-di-GMP production whereas assistance of RcsA to RcsB greatly enhanced this inhibiting effect. Further gene regulation experiments showed that RcsB in assistance of RcsA tightly bound to corresponding promoter-proximal regions to achieve transcriptional repression of *hmsCDE*, *hmsT* and *hmsHFRS* and, meanwhile, RcsAB positively regulated *hmsP* most likely in an indirect manner. Data presented here disclose that pseudogenization of *rcsA* leads to dramatic remodeling of RcsAB-dependent *hms* gene expression between *Y. pestis* and its progenitor *Y. pseudotuberculosis*, enabling potent production of *Y. pestis* biofilms in fleas.

Y*ersinia pestis* is an extremely virulent pathogen causing severe invasive infections mainly manifested as bubonic plague in lymph nodes, septicemic plague in blood vessels, and pneumonic plague in lungs. *Y. pestis* is potent to synthesize biofilms, which are a population of bacterial colonies embedded in self-produced matrix^1^,^2^,^3^. Formation of attached *Y. pestis* biofilms in flea gut is important for flea-borne transmission of this pathogen^1^,^2^,^3^.

*Y. pestis* biofilm matrix is primarily composed of poly-B-1,6-N-acetylglucosamine exopolysaccharide^1^,^2^,^3^. The *hmsHFRS* operon is responsible for biosynthesis and translocation of biofilm exopolysaccharide through cell envelope^4^,^5^. HmsR and HmsS are located in inner membrane, whereas HmsH and HmsF are outer-membrane proteins^5^. HmsR has four transmembrane domains plus a cytoplasmic glycosyltransferase domain, while HmsS has two transmembrane domains; HmsR and HmsS form an enzymatic complex responsible for exopolysaccharide biosynthesis^4^,^6^,^7^. HmsH acts as a porin with *β*-barrel structure, and HmsF functions as a polysaccharide deacetylase; these two proteins form a complex for modification/export of partially deacetylated exopolysaccharide through outer membrane^5^,^8^.

The 3′,5′-cyclic diguanosine monophosphate (c-di-GMP), a small-molecule second messenger promoting exopolysaccharide biosynthesis, is produced from guanosine triphosphate by GGDEF-domain-containing diguanylate cyclases and degraded by EAL-domain-containing phosphodiesterases^9^. *Y. pestis* produces a total of two diguanylate cyclases HmsT and HmsD, and both of them are required for c-di-GMP biosynthesis and biofilm formation^10^,^11^. Although expression of both HmsT and HmsD is up-regulated in flea gut and upon temperature shift from 37°C (as in warm-blooded hosts) to 26°C (in flea gut), HmsD plays a major role in biofilm formation in fleas while the predominant effect of HmsT is on *in vitro* biofilm formation^11^. The *hmsD* gene is a member of the three-gene operon *hmsCDE*. HmsD is a trans-inner-membrane protein composed of three distinct domains, namely a periplasmic sensor domain, an HAMP signal converter domain and a cytoplasmic output GGDEF domain^12^,^13^. The periplasmic protein HmsC senses environmental signals and then interacts with HmsD periplasmic domain, which affects HmsD stability and thereby regulates cellular c-di-GMP levels^12^,^13^. In addition, *Y. pestis* expresses the sole c-di-GMP-specific phosphodiesterase HmsP, which is responsible for degradation of c-di-GMP and therefore has an inhibiting effect on biofilm formation^7^,^14^.

The *hmsHFRS* orthologs can be found in several bacterial species^15^, including the genetically close *pgaABCD* operon in *Escherichia coli*^16^. c-di-GMP binds to PgaC and PgaD (homologues of HmsR and HmsS, respectively), which stabilizes the PgaCD enzymatic complex and thereby activates its glycosyltransferase activity to produce exopolysaccharide^17^. Without c-di-GMP binding, PgaD fails to interact with PgaC and both of them are subject to proteolysis^17^. *Y. pestis* might employ the conserved c-di-GMP-HmsRS association mechanism to control exopolysaccharide production.

The *Enterobacteriaceae* Rcs phosphorelay system is an atypical two-component regulatory system composed of three proteins, RcsB, RcsC and RcsD^18^. RcsC and RcsD are membrane-bound proteins, while RcsB is a cytoplasmic one. RcsC acts as a sensor kinase catalyzing autophosphorylation of RcsD and RcsB, and the resulting phosphate group is then transferred to RcsD and finally to RcsB. Phosphorylated RcsB (RcsB-p) acts as a transcriptional regulator alone or upon binding with an auxiliary protein RcsA. The RcsAB complex recognizes a consensus box sequence TAAGAAT-ATTCTTA, which is a 7-7 invert repeat, within the promoter-proximal regions of its target genes mainly including those responsible for exopolysaccharide biosynthesis, flagellar mobility, and Rcs autoregulation (Table S1, and Fig S1).

The biofilm formation of *Y. pestis* and its genetically very closed progenitor *Y. pseudotuberculosis* is negatively regulated by the Rcs phosphorelay system^19^,^21^. The *rcsA* gene is inactivated in *Y. pestis* due to a 30 bp duplication insertion in its coding region, and replacing the *rcsA* pseudogene with functional *rcsA* allele of *Y. pseudotuberculosis* strongly represses *Y. pestis* biofilm formation and essentially abolished flea blockage^19^,^21^. The conversion of *rcsA* to a pseudogene during evolution from *Y. pseudotuberculosis* to *Y. pestis* is most likely a case of positive Darwinian selection^19^,^21^.

The present work discloses that the RcsAB complex acts as a major repressor of *Y. pestis* biofilm formation through directly repressing transcription of *hmsCDE*, *hmsT* and *hmsHFRS* meanwhile positively regulating *hmsP* in an indirect manner. The above results denote dramatic remodeling of biofilm-related *hms* gene expression between and *Y. pestis* and its progenitor *Y. pseudotuberculosis* due to adaptive pseudogenization of a regulatory gene *rcsA*.

## Results

### Bacterial strains and their biofilm phenotypes

**Figure 1:**
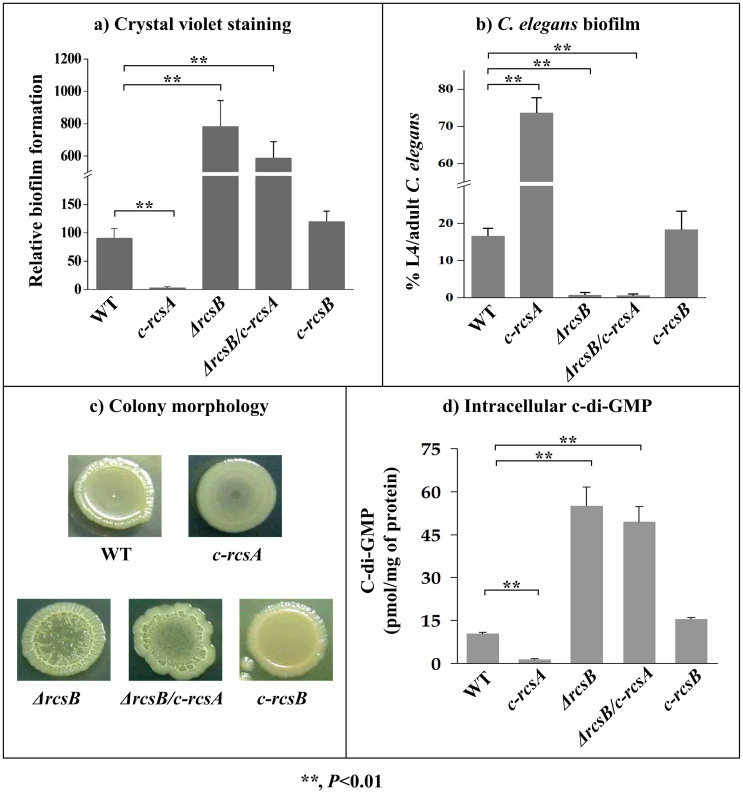
Involvement of RcsAB in biofilm/c-di-GMP production. Crystal violet staining of *in vitro* biofilm masses (a), *C. elegans* biofilms (b), bacterial colony morphology (c), and bacterial intracellular c-di-GMP concentration (d) were determined.

Transformation of pACYC184-*rcsA* into WT (wild-type, RscB+ and RcsA-) generated the *rcsA-*complemented strain *c-rcsA* (RscB+ and RcsA+), which led to a considerable decrease in c-di-GMP/biofilm production (Fig. 1).

Deletion of *rcsB* from WT generated the *rcsB*-null strain *ΔrcsB* (RscB- and RcsA-), and further transformation of pACYC184-*rcsA* into *ΔrcsB* generated another *rcsA*-complemented strain *ΔrcsB/c-rcsA* (RscB- and RcsA+); compared to WT, both *ΔrcsB* and *ΔrcsB/c-rcsA* gave significantly enhanced c-di-GMP/biofilm production (Fig. 1).

Transformation of pACYC184-*rcsB* into *ΔrcsB* generated the *rcsB-*complemented strain *c-rcsB* (RscB+ and RcsA-), which had a biofilm/c-di-GMP production phenotype very similar to WT (Fig. 1).

Taken together, RcsB alone (but not RcsA alone) has an inhibiting effect on biofilm/c-di-GMP production, whereas assistance of RcsA to RcsB greatly enhances this inhibiting effect.

### Regulation of hmsCDE, hmsT, hmsHFRS and hmsP by RcsAB

RcsAB box-like sequences could be found within the promoter-proximal regions of *hmsCDE*, *hmsT* and *hmsHFRS*, indicating that they might serve as direct RcsAB targets (Table S3), which promoted us to elucidate RcsAB-dependent expression of these candidate genes. *hmsP* was also included in the following gene regulation analyses.

**Figure 2:**
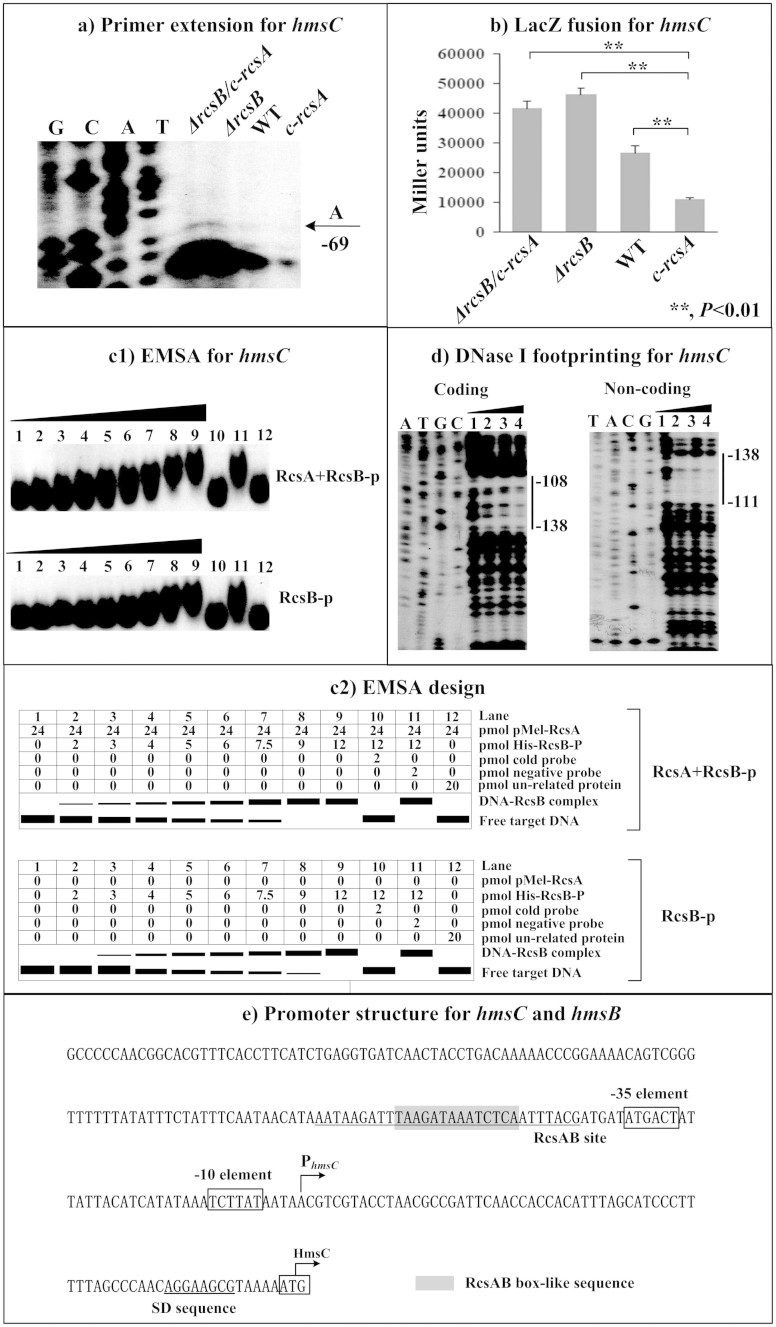
RcsAB-dependent expression of *hmsCDE*. (a) Primer extension. The relative mRNA levels of *hmsC* in indicated strains were determined by primer extension. The Sanger sequence ladders (lanes G, C, A, and T) and the primer extension products of *hmsC* were analyzed with an 8 M urea-6% acrylamide sequencing gel. The transcription start site of *hmsC* was indicated by arrow with nucleotide A, and the minus number under arrow indicated the nucleotide position upstream of *hmsC* start codon. (b) LacZ fusion. The *hmsC*:*lacZ* transcriptional fusion vector was transformed into in indicated strains, and then *hmsC* promoter activities (miller units of β-galactosidase activity) were determined in bacterial cellular extracts. (c) EMSA. The radioactively labeled DNA fragments were incubated with indicated purified proteins and then subjected to a native 4% polyacrylamide gel electrophoresis. (d) DNase I footprinting. Labeled coding or non-coding DNA probes were incubated with indicated purified proteins and then subjected to DNase I digestion. The digested DNA samples were analyzed in an 8 M urea-6% polyacrylamide gel. The footprint regions were indicated with vertical bars. Lanes C, T, A, and G represented Sanger sequencing reactions. The DNA-binding of His-RcsB-p in presence of MBP-RcsA (involved in EMSA and DNase I footprinting) and that of His-RcsB-p alone (in EMSA) were tested. (d) Promoter structure. Shown were with translation/transcription starts, core promoter −10 and −35 elements, SD sequences, RcsAB sites, and RcsAB box-like sequences.

**Figure 3:**
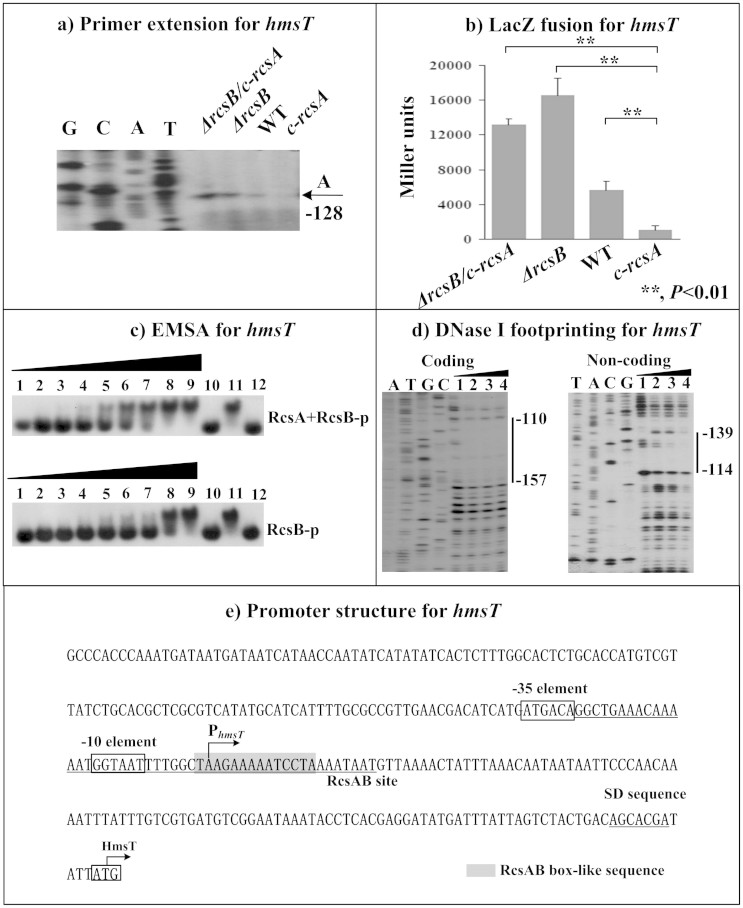
RcsAB-dependent expression of *hmsT*. Primer extension (a), LacZ fusion (b), EMSA (c), DNase I footprinting (d) experiments were performed as described in Fig 2.

**Figure 4:**
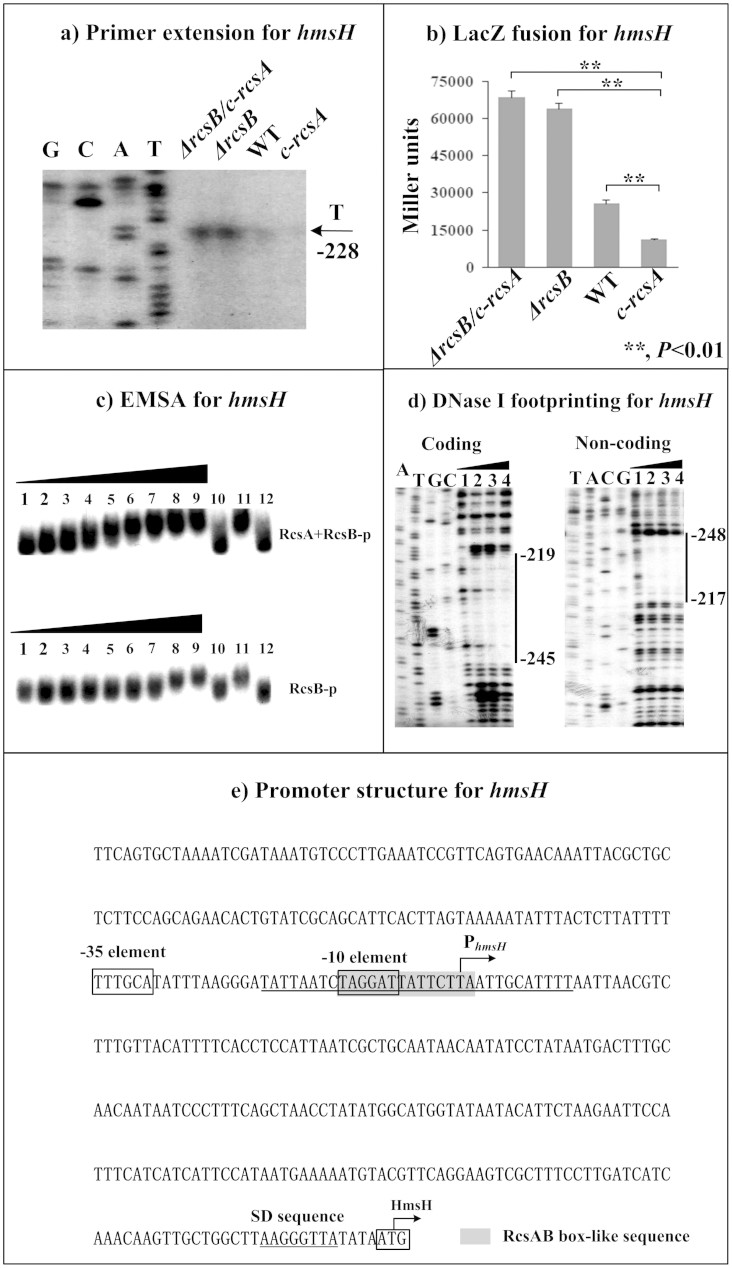
RcsAB-dependent expression of *hmsHFRS*. Primer extension (a), LacZ fusion (b), EMSA (c), and DNase I footprinting (d) experiments were performed as described in Fig 2.

The primer extension assays indicated the relative mRNA levels of each of *hmsC* (Fig. 2a), *hmsT* (Fig. 3a) and *hmsH* (Fig. 4a) in the below four strains showed the following tendency: *c-rcsA* <WT <*ΔrcsB* ≈ *ΔrcsB/c-rcsA*. This observation was further confirmed by determination of the promoter activities of the above four genes by LacZ fusion (Fig. 2b, Fig. 3b, and Fig. 4b). As determined by electrophoretic mobility shift assay (EMSA), His-RcsB-p alone or mixed with excess MBP-RcsA could bind to the promoter-proximal region of each of *hmsC* (Fig. 2c), *hmsT* (Fig. 3c) and *hmsH* (Fig. 4c) in a dose-dependent manner; moreover, addition of excess RcsA could improve DNA-binding activity of RcsB-p. Further DNase I footprinting experiments showed that His-RcsB-p in presence of MBP-RcsA protected a single upstream region of each of *hmsC* (Fig. 2d), *hmsT* (Fig. 3d) and *hmsH* (Fig. 4d). The above observations indicated that RcsB-p in assistance of RcsA tightly bound to the corresponding promoter-proximal regions to achieve transcriptional repression of *hmsCDE*, *hmsT* and *hmsHFRS*.

**Figure 5:**
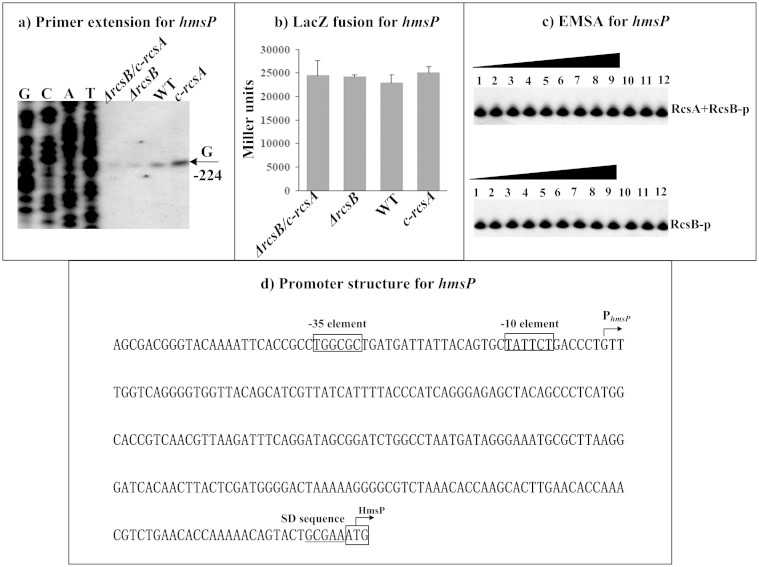
RcsAB-dependent expression of *hmsP*. Primer extension (a), LacZ fusion (b), and EMSA (c) experiments were performed as described in Fig 2.

By contrast, the relative mRNA levels (determined by primer extension, Fig. 5a) of *hmsP* showed the following tendency: *c-rcsA*> WT> *ΔrcsB* ≈ *ΔrcsB/c-rcsA*, which was further validated by quantitative RT-PCR (data not shown). However, LacZ fusion assay (Fig. 5b) indicated that RcsAB had no regulatory effect on promoter activity of *hmsP*. In addition, both EMSA (Fig. 5c) and DNase I footprinting (data not shown) indicated no association between RcsAB and *hmsP* upstream DNA. Therefore, RcsAB positively regulated *hmsP* most likely in an indirect manner.

### Organization of RcsAB-dependent promoters

Transcription starts determined by primer extension were considered as transcribed promoters for indicated genes and, accordingly, core promoter −10 and −35 elements could be predicted. Each of *hmsCD* (Fig. 2e), *hmsT* (Fig. 3e), *hmsP* (Fig. 4e) and *hmsHFRS* (Fig. 5d) had a single transcribed promoter. It should be noted that all the above data were consistent with our previous report on regulation of *hms* genes by *Y. pestis* ferric uptake regulator Fur^20^.

The footprints determined by DNase I footprinting were considered as RcsAB sites for *hmsCDE*, *hmsT*, and *hmsH*; as expected, RcsAB box-like sequences (Table S3) could be found within all these RcsAB sites. The organization of RcsAB-dependent promoters of *hmsCDE* (Fig. 2e), *hmsT* (Fig. 3e), *hmsHFRS* (Fig. 4e), and *hmsP* (Fig. 5d) was constructed with translation/transcription starts, core promoter −10 and −35 elements, predicted Shine-Dalgarno (SD) sequences for ribosomal binding, RcsAB sites, and RcsAB box-like sequences.

## Discussion

**Figure 6:**
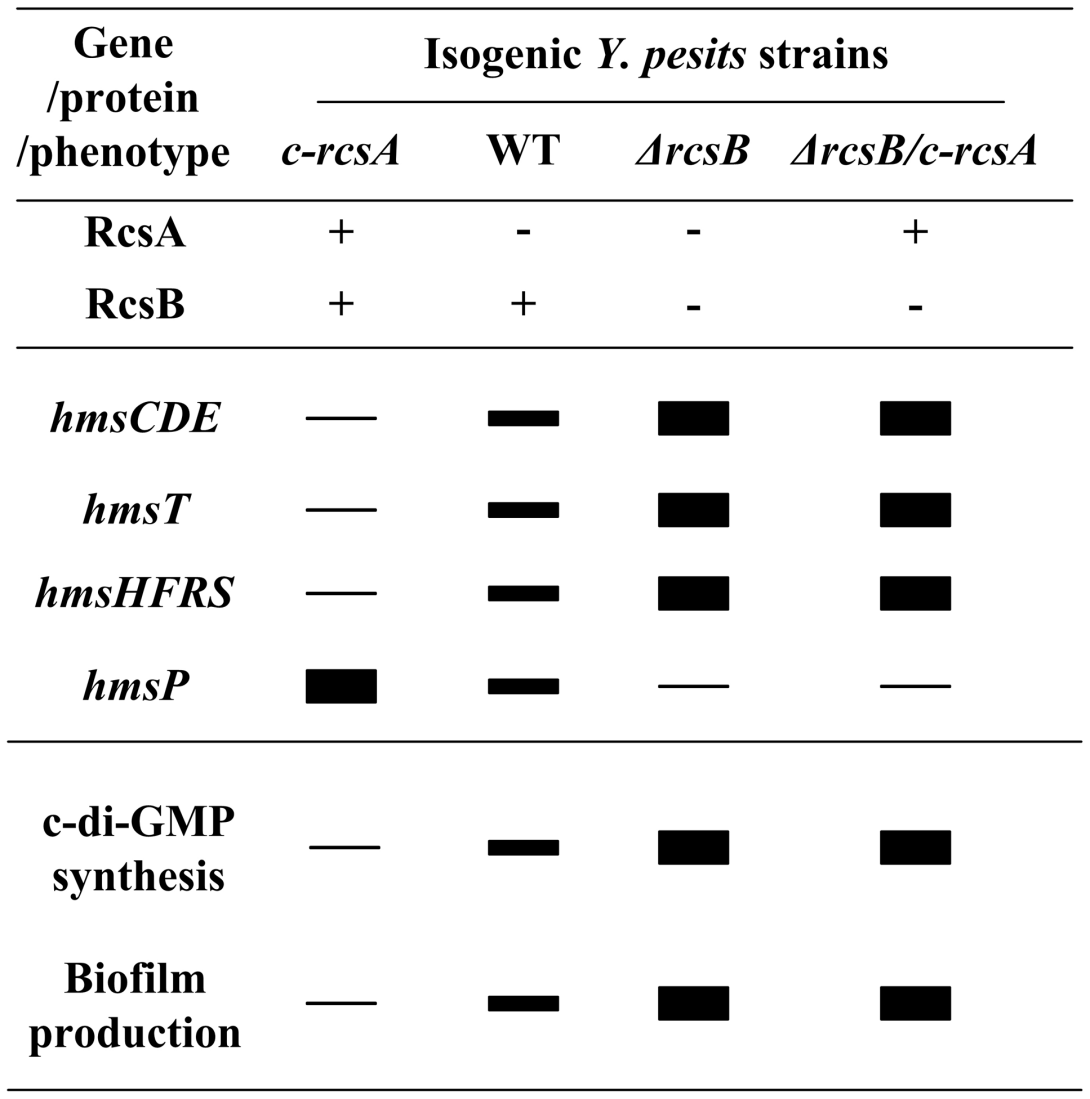
RcsAB-dependent gene expression and phenotypes. Shown were relative mRNA levels of each of *hmsCDE*, *hmsT*, *hmsHFRS* and *hmsP* in different isogenic *Y. pestis* strains*,* as well as relative potencies to produce c-di-GMP/biofilm of these strains.

Transcriptional repression of genes for biofilm exopolysaccharide biosynthesis by RcsB with assistance of its auxiliary protein RcsA has been characterized in several bacterial species (Table S1). The present work confirms RcsAB-mediated tight inhibition of *Y. pestis* c-d-GMP/exopolysaccharide/biofilm production by using a set of isogenic strains of *Y. pestis* biovar *Microtus*, namely WT (RscB+ and RcsA-), *c-rcsA* (RscB+ and RcsA+), *ΔrcsB* (RscB- and RcsA-), and *ΔrcsB/c-rcsA* (RscB- and RcsA+). RcsAB acts as a major repressor of *Y. pestis* biofilm formation through directly repressing transcription of biofilm-enhancing genes *hmsCDE*, *hmsT* and *hmsHFRS* and meanwhile positively regulating biofilm-enhancing one *hmsP* in an indirect manner. RcsB in absence of RcsA does have residual regulatory effects on biofilm formation and *hms* gene expression and, moreover, RcsB-dependent regulation is greatly increased with assistance of RcsA, which was consistent with previous results^19^,^21^,^22^. The above regulatory circuit leads to different expression levels of each of *hmsCDE*, *hmsT*, *hmsHFRS* and *hmsP* in the above isogenic strains and thus distinct potencies of these strains to produce c-di-GMP/biofilm (summarized in Fig. 6).

*Y. pseudotuberculosis* (RscB+ and RcsA+, analogous to *Y. pestis* strain *c-rcsA* in this study] has a biofilm^-^ phenotype in fleas^19^,^21^,^22^. In *Y. pseudotuberculosis*, biosynthesis of HmsCDE, HmsT, and HmsHFRS is tightly inhibited while HmsP is allowed to express. The pseudogenization of *rcsA* leads to inability of RcsAB complex in *Y. pestis*, which in turn alleviates RcsAB-mediated inhibition of expression of *hmsCDE*, *hmsT*, and *hmsHFRS*. As a prerequisite of potent *Y. pestis* biofilm formation, the adaptive pseudogenization of *rcsA* results in dramatic remodeling of *hms* gene expression patterns between *Y. pseudotuberculosis* and *Y. pestis*, finally enabling *Y. pestis* biofilm formation in fleas and thereby flea-borne transmission of this pathogen.

RcsB, RcsC, and RcsD are still functional in *Y. pesits* and thus, there is residual RcsB-dependent repression of biofilm formation in this bacterium^19^. Preclusion of total inactivation of Rcs phosphorelay during *Y. pestis* evolution might be due to the following reasons: biofilm overproduction if *rcsB* is inactivated would has detrimental effects on flea as vectors as well as on bacterial growth and proliferation; Rcs phosphorelay plays roles during mammalian infections^23^.

As shown previously^23^, RcsAB binds to the promoter-proximal region of *hmsT* to repress *hmsT* transcription. As disclosed in this study, RcsAB inhibits transcription of *hmsCDE*, *hmsT*, and *hmsHFRS* through binding to the promoter-proximal regions of all these direct RcsAB targets. RcsAB sites overlap core promoter -10 elements and transcription start sites of *hmsT* and *hmsHFRS*. Association between RcsAB and the above target promoter regions will block entry of RNA polymerase to inhibit transcription of *hmsT* and *hmsHFRS*, which has been characterized for RcsAB-mediated transcriptional repression of an array of direct target genes in other *Enterobacteriaceae* organisms^24^,^25^,^26^. Notably, the RcsAB site is upstream of promoter −35 element of *hmsCDE* and, thus, inhibitory action of RcsAB on *hmsCDE* transcription appears to be highly unusual, which needs to be further elucidated.

## Methods

### Bacterial strains

The wild-type *Y. pestis* *Microtus* strain 201 (WT) is avirulent to humans but highly virulent to mice^27^. The partial coding region of each indicated gene was replaced by a kanamycin resistance cassette by using the one-step inactivation method based on the lambda phage recombination system^28^, to generate the corresponding mutant of *Y. pestis* (Table 1). For *in trans* complementation, a PCR-generated DNA fragment containing the coding region of each indicated gene together with its promoter-proximal region and transcriptional terminator-proximal region was cloned into the cloning vector pACYC184 (GenBank accession no. X06403), and the resulting recombinant vector was transformed into each indicated *Y. pestis* strain lack of the corresponding functional gene, generating the corresponding complemented mutant (Table 1). All the primers designed in this study are listed in Table S2.

### Bacterial growth and RNA isolation

Overnight cell cultures in the Luria-Bertani (LB) broth with an optical density (OD_620_) of about 1.0 were diluted 1:50 into 18 ml of fresh LB broth for further cultivation at 26°C with shaking at 230 rpm to reach middle stationary phases (an OD_620_ of 0.8 to 1.2), followed by cell harvest for further gene regulation or phenotypic assays. Immediately before bacterial harvest for RNA isolation, double-volume of RNAprotect reagent (Qiagen) was mixed with one-volume of cell culture, and total RNA was extracted using TRIzol Reagent (Invitrogen). RNA quality was monitored by agarose gel electrophoresis, and RNA quantity was determined by spectrophotometry.

### Primer extension assay

As described in our previous studies^29^,^30^, a 5′-^32^P-labeled oligonucleotide primer complementary to a portion of the RNA transcript of each indicated gene was employed to synthesize cDNAs from total RNA templates using Promega Primer Extension System. Sequence ladders were prepared with the same 5′-^32^P-labeled primers using AccuPower & Top DNA Sequencing Kit (Bioneer). Radioactive species were detected by autoradiography. If different *Y. pestis* strains were involved in a single experiment, equal amounts of the total RNA samples were used as the starting materials. The relative mRNA level was determined with the observed band intensity of the primer extension product of each target gene. The 5′-terminus of RNA transcript (i.e., transcription start) of each target gene was mapped according to the size of primer extension product.

### LacZ fusion and β-galactosidase assay

A promoter-proximal DNA region of each indicated gene was cloned into the low-copy-number transcriptional fusion vector pRW50^31^ that harbors a promoterless *lacZ* reporter gene. *Y. pestis* strains transformed with the recombinant plasmid or the empty pRW50 (negative control) were grown to measure β-galactosidase activity in cellular extract using β-Galactosidase Enzyme Assay System (Promega)^29^,^30^.

### Protein expression and purification

The entire coding region of *Y. pseudotuberculosis rcsA* or *Y. pestis* *rcsB* was cloned into plasmid pMAL-c4X (Invitrogen)^23^ or pBADMyc-His A (New England Biolabs)^23^, respectively. The wild-type *Y. pestis* strain KIM6+ and the *rcsB* null mutant of KIM6+ were employed as host cells for expression of maltose-binding protein (MBP)-tagged RcsA (MBP-RcsA) and 6 × His-tagged RcsB (His-RcsB), respectively^23^. His-RcsB and MBP-RcsA were purified under native conditions using Ni-NTA Agarose Column (Qiagen) and Amylose Agarose Column (New England Biolabs), respectively^23^. Each purified protein was dialyzed and then concentrated to a concentration of about 0.1 mg/ml in phosphate buffered saline (pH 8.0) containing 20% glycerin.

### EMSA

Each indicated 5′-^32^P-labeled target DNA fragment was incubated with increasing amounts of purified His-RcsB, or with increasing amounts of purified His-RcsB with addition of 24 pmol of purified MBP-RcsA, for 30 min at room temperature in a binding buffer^29^,^30^. To achieve RcsB phosphorylation, 25 mM fresh acetyl phosphate was incubated for 30 min with His-RcsB in the binding buffer, before labeled DNA probes were added. The resulting reactions were subjected to a native 4% (w/v) polyacrylamide gel electrophoresis. Each EMSA experiment included three controls, namely, cold probe as the specific DNA competitor (the same promoter-proximal DNA region unlabeled), negative probe as the nonspecific DNA competitor (the unlabeled coding region of the 16S rRNA gene), and nonspecific protein competitor (rabbit anti-F1-protein polyclonal antibodies)^29^,^30^. Detection of sequencing and radioactive species was as above.

### DNase I footprinting

For DNase I footprinting^29^,^30^, the target DNA fragment with a single ^32^P-labeled end was incubated with increasing amounts of purified His-RcsB-p with addition of 24 pmol of purified MBP-RcsA, which was followed by partial digestion of RQ1 RNase-Free DNase I (Promega). The digested DNA samples were purified and analyzed in an 8 M urea-6% polyacrylamide gel. Detection of sequencing and radioactive species was as above. Footprints were identified by comparison with sequence ladders.

### Biofilm and c-di-GMP assays

As described in our previous study^32^, three different methods were used to detect *Y. pestis* biofilms. First, *in vitro* biofilm masses, attached to well walls when bacteria were grown in polystyrene microtiter plates, were stained with crystal violet. Second, percentages of fourth-stage larvae and adults (L4/adult) of *C. elegans* after incubation of nematode eggs on *Y. pestis* lawns, negatively reflecting bacterial ability to produce biofilms, were determined. Third, rugose colony morphology of bacteria grown on LB agar plates, positively reflecting bacterial ability to synthesize exopolysaccharide, was observed. In addition, intracellular c-di-GMP levels were determined by a chromatography-coupled tandem mass spectrometry (HPLC-MS/MS) method as described in our previous study^20^.

### Experimental replicates and statistical methods

For LacZ fusion, crystal violet staining of biofilms, and determination of L4/adult nematodes or c-di-GMP, experiments were performed with at least three independent bacterial cultures/lawns, and values were expressed as mean ± standard deviation. Paired Student's *t-*test was performed to determine statistically significant differences; *P* <0.01 was considered to indicate statistical significance. For primer extension and colony morphology observation, shown were representative data from at least two independent bacterial cultures.

## Supplementary Material

Supplementary Information Supplementary Information

## Figures and Tables

**Table 1:** *Y. pestis* strains involved in gene deletion and complementation

Strain	Functional (+) or inactivated (-)					Feature	Reference
	*rcsA*	*rcsB*	*hmsD*	*hmsT*	*hmsS*		
WT	−	+	+	+	+	The wild-type *Y. pestis* biovar *Microtus* strain 201.	[27]
*c-rcsA*	+	+	+	+	+	The vector pACYC184-*rcsA*^#^ was introduced into WT.	This study
*ΔrcsB*	−	−	+	+	+	The base pairs 211 to 418 of *rcsB* gene was deleted from WT.	This study
*c-rcsB*	−	+	+	+	+	The vector pACYC184-*rcsB* was introduced into *ΔrcsB*.	This study
*ΔrcsB/c-rcsA*	+	−	+	+	+	The vector pACYC184-*rcsA*^#^ was introduced into *ΔrcsB*.	This study
*ΔhmsTΔhmsD*	−	+	−	−	+	A reference c-di-GMP- strain. The base pairs -4 to 1179 of *hmsT* gene was deleted from WT, and then the base pairs 41 to 1238 of *hmsD* gene was deleted from *ΔhmsT*.	This study
*ΔhmsS*	−	+	+	+	−	A reference biofilm- strain. The base pairs 146 to 468 of *hmsS* was deleted from WT.	[20]

*#*: functional *Y. pseudotuberculosis rcsA.*
