# Supplementary material for: RcsAB is a major repressor of Yersinia biofilm development through directly acting on hmsCDE, hmsT, and hmsHFRS
Source: Sci Rep. 2015 Apr 1;5:9566. doi: 10.1038/srep09566 (PMC4381331; doi:10.1038/srep09566)
Supplement: Supplementary Information [file srep09566-s1.doc]

**RcsAB is a major repressor of *Yersinia* biofilm development through directly acting on *hmsCDE*, *hmsT*, and *hmsHFRS***

Nan Fang, Huiying Yang, Haihong Fang, Lei Liu, Yiquan Zhang, Li Wang, Yanping Han, Dongsheng Zhou, Ruifu Yang

**Table S1. Collection of RcsAB box-like sequences**

| **Bacterium** | **Gene** | **RcsAB box-like sequence** | **Reference** |
| --- | --- | --- | --- |
| **Exopolysaccharide synthesis and regulation** | | | |
| *Escherichia coli/Shigella* | *wza* | TAAAGAAACTCCTA |  |
|  | *yjbE* | TGAGGTTAATCCTA |  |
|  | *galF* | TAAGATTATTCTCA |  |
| *Salmonella enterica* | *tviA* | TAGGAATATTCTTA |  |
| *Klebsiella pneumoniae* | *galF* | TAAGGAAATTCTGA |  |
| *Y. pseudotuberculosis*  */Y.pestis* | *hmsH* | TAGGATTATTCTTA | This study |
|  | *hmsT* | TAAGAAAAATCCTA |  |
|  | *hmsC* | TAAGATAAATCTCA |  |
| *Erwinia amylovora* | *amsG* | TGAGAATAATCTTA |  |
| *Pantoea stewartii* | *cpsA* | TGGAATAAATCTGA |  |
| **Mobility** | | | |
| *E. coli* | *flhD* | TAGGAAAAATCTTA |  |
| *S. enterica* |  | TAGGAAAAATCTTA | Predicted |
| *Y. enterocolitica* |  | TAGGAATAATCCTA | Predicted |
| *Y. pseudotuberculosis* |  | TAGGAATATTCCTA | Predicted |
| **RcsA** | | | |
| *E. coli/Shigella* | *rcsA* | TAAGGATTATCCGA |  |
| *S. enterica* |  | TAAGGTTTATCCGA |  |
| *K. pneumoniae* |  | TAAGGAAATTCTGA |  |
| *Y. pseudotuberculosis* |  | TACGGATTTTCCGA | Predicted |
| *E. amylovora* |  | TAAGAATAGTCCTA |  |

*Shigella* species, *K. pneumoniae*, and *Y. pestis* are negative for motility due to the absence of *flhDC* or the presence of nonfunctional *flhDC*. *rcsA* is inactivated in *Y. pestis*.


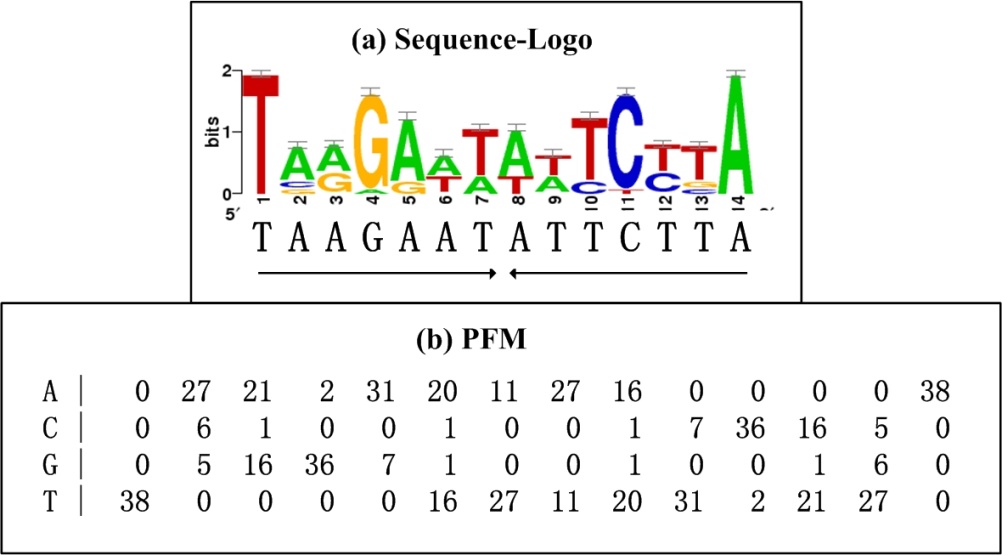


**Fig. S1 RcsAB consensus constructs.** (a) The sequence logo of aligned RcsAB box-like sequences (Table S3). (b) The position frequency matrix (PFM) denoting the frequency of each nucleotide (raw) at each nucleotide position (column).

**Table S2 Primer used in this study**

| **Target**  **gene** | **Primers (5'-3'; F/R)** |
| --- | --- |
| **Mutant construction** | |
| *rcsB* | CTCTATGCCAGGGGATAAGTATGGTGATGGCATCACATTAAGATTGCAGCATTACACG/GGTGACAAACGCTTGTCACCGTAACCATTGGCACTGATTTTGTAACGCACTGAGAAGC |
| *hmsT* | AAATACCTCACGAGGATATGATTTATTAGTCTACTGACAGCACGA AGATTGCAGCATTACACG/ATCATTAAAAATAATAACTGGCATAGAGCGTCCGTATGTTCAGTG TGTAACGCACTGAGAAGC |
| *hmsD* | ATGAAGCCGAAAAAAAATCACAATAGCGCAGAAAAACTCCAGATTGCAGCATTACACGTC/CTATCCTAAACTTTCTGTGTTAGTCGTATTCGGCTGATAGTGTAACGCACTGAGAAGC |
| **Complementation of the mutant** | |
| *rcsA* | GCG***GTCGAC***cggcagcacagcgtaaac/ *GCG****GGATCC***GGTCACTACTCAGGCAAGAAAG |
| *rcsB* | GCG***GTCGAC***GAGCGGCGAATTTTATCTGAAC/ *GCG****GGATCC***GTTTGCGGCAGTTGAATCAC |
| **Primer extension** | |
| *hmsH* | /TATTGTTGCAAAGTCATTATAGGAT |
| *hmsT* | /GGTATTTATTCCGACATCACGAC |
| *hmsC* | /AGTAGCGGTAGTCATTTTTACG |
| *hmsP* | /CCATCGAGTAAGTTGTGATCC |
| **LacZ reporter fusion** | |
| *hmsH* | GCGGGATCCACTTTGCTGAAGACTTGTCACG/ GCGAAGCTTCCGCCATAGCAGGATTAACG |
| *hmsT* | GCGGAATTCGCCCAGTACAGGTAACAAGG/ GCGGGATCCCTGATCGTAGGAGTGGCTATTC |
| *hmsC* | TCTGGATCCCTTACTGGTTGCTATTGCC/ TCTAAGCTTGAGGTTCATGATGTTCATCA |
| *hmsP* | GCGGGATCCAGCGATGGTAGAAGTGAATCAG/ GCGAAGCTTTTGCGATACTCTAATGGAAGGC |
| **EMSA** | |
| *hmsH* | ACTTTGCTGAAGACTTGTCACG/ CCGCCATAGCAGGATTAACG |
| *hmsT* | GCCCAGTACAGGTAACAAGG/CTGATCGTAGGAGTGGCTATTC |
| *hmsC* | CTTACTGGTTGCTATTGCC/GAGGTTCATGATGTTCATCA |
| *hmsP* | GCGATGGTAGAAGTGAATCAG/TAGGAACGCCATTTCGCAG |
| **DNase I footprinting** | |
| *hmsH* | ACACTGTATCGCAGCATTCAC/GGGATTATTGTTGCAAAGTCA |
| *hmsT* | tttggcactctgcaccatg/CGTGAGGTATTTATTCCGACA |
| *hmsC* | ACGGCACGTTTCACCTTC/AAGGGATGCTAAATGTGGTG |

**Table S3 Prediction of *hmsH, hmsT*, and *hmsC* as direct RcsAB targets**.

| **Gene** | **Strand** | **Start** | **End** | **RcsAB box-like sequence** | **Score** |
| --- | --- | --- | --- | --- | --- |
| *hmsH* | D | -240 | -227 | TAGGATTATTCTTA | 13.1 |
| *hmsT* | D | -130 | -117 | TAAGAAAAATCCTA | 12.0 |
| *hmcC* | D | -129 | -116 | TAAGATAAATCTCA | 10.4 |
| *hmsP* | Not applicable | | | | |

Known or predicted RcsAB box-like sequences in *Enterobacteriaceae* were collected (Table S1) and aligned to generate two RcsAB consensus constructs (Fig. S1) by using the ‘*matrices-consensus*’ tool : an updated RcsAB box sequence TAAGAAT-ATTCTTA (a 7-7 invert repeat), and a PFM recording the position-dependent frequency of each nucleotide. A PFM described a sequence motif more precisely a box (string) sequence, because a PFM was modeled from a set of box-like sequences. The PFM representing conserved DNA signals recognized by RcsAB was used for consensus matching within the 300bp upstream regions of *hmsT*, *hmsCDE*, *hmsHFRS*, and *hmsP* by using the ‘*matrices-paster*’ tool . This analysis predicted the potential RcsAB box-like sequences with score values (see the above table). Higher score value indicated higher probability of regulator-target promoter recognition. When a frequently used cutoff value of seven were used for the score values, the above computational promoter analysis identified the RcsAB box-like sequences within the promoter-proximal region of *hmsH, hmsT*, and *hmsC*, suggested that RcsAB could recognize these corresponding promoter regions for transcriptional regulation.

**References**

1. Wehland M, Bernhard F: **The RcsAB box. Characterization of a new operator essential for the regulation of exopolysaccharide biosynthesis in enteric bacteria**. *J Biol Chem* 2000, **275**(10):7013-7020.

2. Stout V: **Identification of the promoter region for the colanic acid polysaccharide biosynthetic genes in Escherichia coli K-12**. *J Bacteriol* 1996, **178**(14):4273-4280.

3. Ferrieres L, Aslam SN, Cooper RM, Clarke DJ: **The yjbEFGH locus in Escherichia coli K-12 is an operon encoding proteins involved in exopolysaccharide production**. *Microbiology* 2007, **153**(Pt 4):1070-1080.

4. Rahn A, Whitfield C: **Transcriptional organization and regulation of the Escherichia coli K30 group 1 capsule biosynthesis (cps) gene cluster**. *Mol Microbiol* 2003, **47**(4):1045-1060.

5. Carlier AL, von Bodman SB: **The rcsA promoter of Pantoea stewartii subsp. stewartii features a low-level constitutive promoter and an EsaR quorum-sensing-regulated promoter**. *J Bacteriol* 2006, **188**(12):4581-4584.

6. Wehland M, Kiecker C, Coplin DL, Kelm O, Saenger W, Bernhard F: **Identification of an RcsA/RcsB recognition motif in the promoters of exopolysaccharide biosynthetic operons from Erwinia amylovora and Pantoea stewartii subspecies stewartii**. *J Biol Chem* 1999, **274**(6):3300-3307.

7. Francez-Charlot A, Laugel B, Van Gemert A, Dubarry N, Wiorowski F, Castanie-Cornet MP, Gutierrez C, Cam K: **RcsCDB His-Asp phosphorelay system negatively regulates the flhDC operon in Escherichia coli**. *Mol Microbiol* 2003, **49**(3):823-832.

8. Ebel W, Trempy JE: **Escherichia coli RcsA, a positive activator of colanic acid capsular polysaccharide synthesis, functions To activate its own expression**. *J Bacteriol* 1999, **181**(2):577-584.

9. Virlogeux I, Waxin H, Ecobichon C, Lee JO, Popoff MY: **Characterization of the rcsA and rcsB genes from Salmonella typhi: rcsB through tviA is involved in regulation of Vi antigen synthesis**. *J Bacteriol* 1996, **178**(6):1691-1698.

10. Stout V, Torres-Cabassa A, Maurizi MR, Gutnick D, Gottesman S: **RcsA, an unstable positive regulator of capsular polysaccharide synthesis**. *J Bacteriol* 1991, **173**(5):1738-1747.

11. Bernhard F, Poetter K, Geider K, Coplin DL: **The rcsA gene from Erwinia amylovora: identification, nucleotide sequence, and regulation of exopolysaccharide biosynthesis**. *Mol Plant Microbe Interact* 1990, **3**(6):429-437.

12. van Helden J: **Regulatory sequence analysis tools**. *Nucleic Acids Res* 2003, **31**(13):3593-3596.
